# Language Experience with a Native-Language Phoneme Sequence Modulates the Effects of Attention on Cortical Sensory Processing

**DOI:** 10.3389/fnins.2017.00569

**Published:** 2017-11-06

**Authors:** Monica Wagner, Jungmee Lee, Francesca Mingino, Colleen O'Brien, Adam Constantine, Valerie L. Shafer, Mitchell Steinschneider

**Affiliations:** ^1^Department of Communication Sciences and Disorders, St. John's University, New York, NY, United States; ^2^Department of Communication Sciences and Disorders, University of South Florida, Tampa, FL, United States; ^3^Graduate Center, City University of New York, New York, NY, United States; ^4^Department of Neurology, Albert Einstein College of Medicine, New York, NY, United States

**Keywords:** attention, P1-N1-P2, T-complex, auditory evoked potential (AEP), spectro-temporal features, native-language

## Abstract

Auditory evoked potentials (AEP) reflect spectro-temporal feature changes within the spoken word and are sufficiently reliable to probe deficits in auditory processing. The current research assessed whether attentional modulation would alter the morphology of these AEPs and whether native-language experience with phoneme sequences would influence the effects of attention. Native-English and native-Polish adults listened to nonsense word pairs that contained the phoneme sequence onsets /st/, /sət/, /pət/ that occur in both the Polish and English languages and the phoneme sequence onset /pt/ that occurs in the Polish language, but not the English language. Participants listened to word pairs within two experimental conditions designed to modulate attention. In one condition, participants listened to word pairs and performed a behavioral task to the second word in the pairs (“with task”) and in the alternate condition participants listened to word pairs without performing a task (“without task”). Conditions were counterbalanced so that half the English and Polish subjects performed the “without task” condition as the first testing session and the “with task” condition as the second testing session. The remaining English and Polish subjects performed the tasks in the reverse order. Two or more months separated the testing sessions. Task conditions did not modulate the morphology of the AEP. Attention, however, modulated the AEP by producing a negative shift in the overall waveform. This effect of attention was modulated by experience with a native-language phoneme sequence. Thus, only Polish listeners showed an effect of attention to the native language /pt/ onset when the behavioral task occurred as the second testing session for which attention demands were reduced. This effect began at 400 ms and suggests a mechanism at intermediate stages within auditory cortex that facilitates recognition of the native language for comprehension.

## Introduction

The morphology of the auditory evoked potential (AEP), with peaks termed P1-N1-P2 as recorded at fronto-central sites and the T-complex as recorded over lateral sites, are modulated by spectro-temporal feature changes within spoken words (Martin and Boothroyd, 2000; Giraud et al., 2005; Wagner et al., 2016). As the phoneme sequence of each spoken word constitutes a distinctive and dynamic series of changing spectro-temporal features, the P1-N1-P2 and T-complex responses to each changing feature within the phoneme sequence of the word overlap to form a unique waveform pattern specific for that word (Wagner et al., 2016). These physiological sensitivities to the spectro-temporal features of words are acoustically driven and occur whether or not subjects are familiar with the phonemic sequences. Specifically, our previous studies (Wagner et al., 2012, 2013) revealed that even though English listeners could not distinguish words that began with /pt/ and /pət/ (e.g., /ptima-pətima/ “pteema-peteema”), a Polish phoneme sequence contrast that does not occur at word onset in English, AEP morphologies were highly similar in English and Polish listeners. Thus, the sensory waveform morphology to the /pt/ and /pət/ phoneme sequence onsets reflected the acoustic contrast within the word onsets (e.g., /ptuka/ and /pətuka/), and not their phonemic representations.

These unique waveform patterns are sufficiently reliable in normal subjects that they may have utility for probing deficits in auditory processing (Wagner et al., 2016, 2017). One potential confound for this strategy is that attention also modulates scalp-recorded AEPs. Negative-going waves within the P1-N1-P2 and T-complex components are enhanced in “attend” relative to “non-attend” experimental conditions (e.g., Näätänen et al., 1978, 1980; Woods and Clayworth, 1987). Thus, to further assess the potential utility of these word-specific waveforms, we currently examined the manner upon which attention modulates the morphology of the AEPs specific for each phoneme sequence. Since clinical populations of individuals with auditory processing deficits may also have co-morbid attentional deficits, it is necessary to differentiate modulation of AEPs determined by dynamic spectro-temporal sound features and by attention.

The current experimental design allowed us to examine the effects of attention on the AEPs to a non-target word that prompted the occurrence of a target word. Previous studies examining the effects of attention to auditory stimuli have included designs in which subjects were unaware of when a target might appear in a series of non-targets (Woldorff and Hillyard, 1991; Alain et al., 1993; Nourski et al., 2015). Thus, to perform these tasks participants had to remain vigilant throughout the experiment. As an example, Nourski et al. (2015) examined AEPs and event-related band power (ERBP) derived from electrocorticographic signals recorded directly over lateral temporal cortex. Attention to the target stimulus (relative to the non-target) did not modulate representation of the acoustic attributes of the stimulus occurring within early portions of the neural response. However, attention did modulate later portions of the ERBP, reflecting subsequent sound processing. Hence, we examined the effects of attention on the AEP in response to words that prompt target words to explore neural processes by which one prepares to engage attention to a key word within a passage.

Finally, we examined the effects of attention on AEP responses to familiar and unfamiliar phoneme sequence onsets within non-words in native-English and native-Polish speakers to determine whether language experience modulates the effects of attention on neural speech processing. EEG was recorded while native-English and native-Polish adults listened to same and different non-words pairs within two counterbalanced experimental conditions designed to modulate attention. Non-words within the pairs contained the phoneme sequence onsets /pət/, /st/, /sət/ that occur in both the English and Polish languages and the phoneme sequence onset /pt/ that occurs in only the Polish language. The first word in the word pairs was the focus of the current study.

### Hypothesis

We hypothesized that (1) AEP morphology reflecting processing of the spectro-temporal feature changes within spoken words would remain unchanged for both language groups and experimental conditions (Näätänen, 1990; Nourski et al., 2015) and, (2) consistent with our previous research findings, we predicted attention to affect the AEPs to all phoneme sequences through positive or negative shifts in the waveforms similarly in both English and Polish listeners (Wagner et al., 2013, 2016).

## Materials and methods

### Participants

The EEG was recorded during two testing sessions from each of 48 participants. One of two experimental task conditions designed to modulate attention (“Listening, Without Task” and “Listening, With Task”) was presented during each testing session. The sequence for presentation of task conditions for testing sessions one and two was counterbalanced for two separate groups of 24 participants (12 native-English and 12 native-Polish in each group) (Table 1). Table 1 displays the gender, mean age (and range) at the first testing session, handedness and sequence for task condition presentation for the two groups of 24 subjects tested. The four participant groups are identified in Table 1 by native-language and sequence of condition presentation (i.e., “Attend 2” or “Attend 1”), as described in detail below.

**Table 1:** Displays the gender, mean age (and range) at the first testing session, handedness, and sequence for condition presentation for each of the four groups of participants.

**Subjects**	**12 Native-english Attend 2**	**12 Native-polish Attend 2**	**12 Native-english Attend 1**	**12 Native-polish Attend 1**
Gender	9 female	9 female	9 female	9 female
Age (Range)	27 years (21–37)	29 years (22–36)	28 years (22–39)	30 years (21–38)
Handedness	12 right handed	12 right handed	11 right handed	11 right handed
Session 1	Without task	Without task	With task (Attend)	With task (Attend)
Session 2	With task (Attend)	With task (Attend)	Without task	Without task

*The four participant groups are identified by native-language and sequence for condition presentation. For example, English Attend 2 refers to the English participant group who were presented with the attention task during testing session 2*.

Participants reported no history of speech, language, or academic difficulties. All participants had normal hearing (≤25 dB HL) at the frequencies of 0.5, 1, 2, and 4 kHz. English participants were monolingual speakers who had no exposure to Slavic languages. All parents of these subjects were born in the United States. Polish participants were bilingual Polish-English speakers who came to the US after the age of 15 years with two exceptions. One subject emigrated from Poland to the US at age 10 years and the other came to the US as a young child and first learned English in kindergarten. Both of these subjects performed the syllable identification task in a manner consistent with the Polish language group in that they identified the number of syllables in the words containing the /pt/ and /pət/ onsets with greater than 89% accuracy (see Wagner et al., 2012 for detail).

### Stimuli

Multiple tokens of naturally spoken non-words were presented within same and different pairs. Non-words contained the phoneme sequence onsets /pt/, /pət/, /st/, and /sət/. Phoneme sequences that followed the onsets varied (e.g., /ufɑ/, /imɑ/) and word types (e.g., /pt/ onsets) were matched for rhyme (e.g., /ptufɑ/, /pətufɑ/, /stufɑ/, /sətufɑ/). All non-words contained phoneme sequence onsets that occur in the Polish and English languages with the exception of /pt/, which does not occur in English phonology at word onset. Words were produced by a male bilingual English-Polish speaker, who emigrated from Poland with his family at 6 years of age and became a dominant English speaker, though he attended a Polish school for a full day on Saturdays through high school and only Polish was spoken in the home.

Each word type (e.g., /st/) consisted of 35 different words (e.g., /stimɑ/) that were produced twice. These 70 different tokens were presented twice for a total of 140 presentations per word type. The mean duration (and range) for the word onsets measured from onset to the burst for /t/ were as follows: /sət/ 258 ms (210–325), /st/ 208 ms (159–259), /pət/ 149 ms (123–174), /pt/ 114 ms (84–140). The mean duration (and range) for the whole words for each word type were as follows: /sət/ 698 ms (633–801), /st/ 623 ms (551–728), /pət/ 550 ms (481–671), /pt/ 489 ms (417–604).

### Procedure

Word pairs were presented in free field at 62.5 dB SPL through right and left speakers positioned at an azimuth of 45° from the subject's head (Realistic Minimus-7). Experiments were conducted in a sound-attenuated electrically shielded room. Eprime software (version 1.1) was used for stimulus delivery. An inter-stimulus interval (ISI) of 250 ms separated the words within the pairs and an inter-trial interval (ITI) of 2,000 ms separated the word pairs. EEG epochs time locked to the first word in the word pairs were analyzed. Five hundred and sixty same and different non-word pairs were randomized and separated into seven blocks, which were randomly presented to subjects.

EEGs were recorded as participants listened to non-word pairs during two testing sessions and performed a behavioral task to the second word in the word pairs during one of the two testing sessions. For the “without task” condition, participants were only told to listen to the stimuli. For the “with task” condition, participants listened to the stimuli and performed a syllable identification task to the second word in the pairs. The syllable identification task required participants to push a button labeled “2” on the left of the response box or “3” on the right of the response box to identify whether the second word in the word pairs had two or three syllables. One group of English and Polish participants was presented with the “without task” condition as the first testing session and the “with task” condition as the second testing session. Henceforth, this participant group will be termed “Attend 2” as they attended for task performance during session two. The alternate participant groups that were presented with tasks in a reverse order will be termed “Attend 1” (see Table 1). Each participant was tested on two separate occasions with a mean of 5 months (range 2–16 months) separating the testing sessions to reduce the effects of stimulus and task repetition (Nagy and Rugg, 1989).

#### EEG acquisition and data processing

The EEG was collected using a 64 channel net (Electrical Geodesics, Inc.,) recorded at a sampling rate of 500 Hz, bandpass filtered between 0.1 and 100 Hz and referenced to the vertex electrode Cz. Data was then low pass filtered at 30 Hz using Net Station software (version 4.5.1) and processed using BESA software (version 5.3.7). EEG epochs were 1,000 ms (i.e., 100 ms pre-word and 900 ms post-word onset). An adaptive correction function was used to detect and remove eye blinks and ocular motion (Ille et al., 2002). Remaining artifacts were identified using amplitude and signal gradient criteria. The data was then scanned manually and if an excessive number of trials from a channel contained artifact, data from that channel was interpolated using spherical spline interpolation (Perrin et al., 1989). Data was then re-referenced to an average reference and single trials epochs were exported without averaging. R software (R version 3.3.0) was used for baseline correction, using mean amplitude between −100 to 0 ms.

Each testing session included 140 presentations of each word type, except that one subject from each of the four participant groups was presented with an additional block of stimuli during testing session one. The mean number of epochs accepted for analysis, following artifact correction procedures in response to /pət/, /sət/, /pt/, and /st/, respectively, were: English Attend 2 group, without task 134, 134, 135, and 137; Polish Attend 2-group, without task 135, 133, 135, 137; English Attend 2 group, with task 127, 127, 127, 129; Polish Attend 2 group, with task 133, 132, 131, 134; English Attend 1 group, with task 135, 135, 136, 138; Polish Attend 1 group, with task 132, 132, 133, 135; English Attend 1 group, without task 133, 131, 133, 135; Polish Attend 1 group, without task 129, 130, 129, 132.

#### Statistical analysis

**Figure 1:**
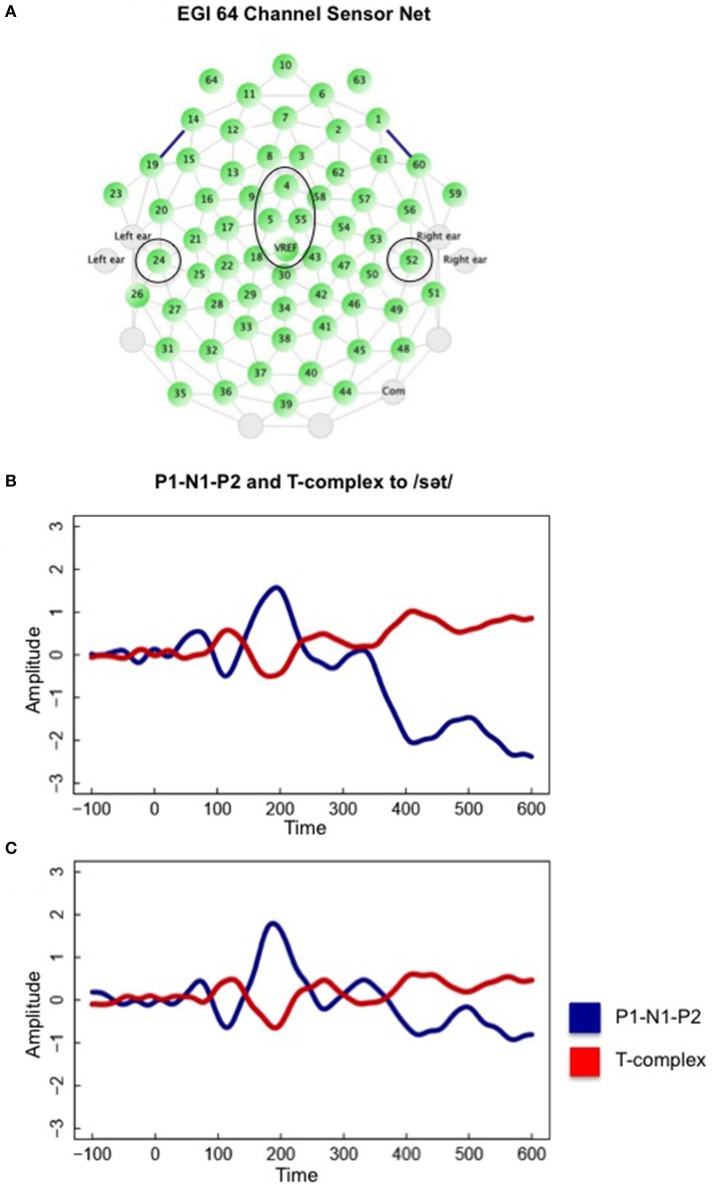
**(A)** The Electrical Geodesics Inc. (EGI) sensor net and fronto-central and posterior temporal electrodes described in the text. Also shown is an overlay of P1-N1-P2 and T-complex to the /s/ onset in the English Attend 1 participant group during **(B)** the “with task” condition and the **(C)** “without task” condition. Notice that T-complex peak latencies are similar to P1-N1-P2 peak latencies in response to a specific phoneme sequence onset.

Mixed effect linear regression model analysis (MEM) (Snijders and Bosker, 2013), which controls for individual subject's mean amplitudes (and slopes) and mean latencies within the fronto-central and posterior temporal AEPs, were used to test the hypotheses. As illustrated in Figure 1A, P1-N1-P2 was defined as an averaged response from four fronto-central (FC) electrode sites, 4, 5, 55, and Cz (VREF), and the T-complex was defined as the averaged response from left and right posterior temporal (PT) electrode sites, 24 and 52. Comparison of P1-N1-P2 and T-complex split epoch averages (Handy, 2005) obtained from these fronto-central and posterior temporal electrodes, has revealed reliable waveform morphologies (Wagner et al., 2017). Alpha level was set at *p* = 0.05 for statistical analyses.

##### Stability of P1-N1-P2 and T-complex morphology

Our prediction that P1-N1-P2 morphology would remain consistent across language groups and tasks that modulated attention was assessed by examining the effects of (1) peak (/sət/ word type: peak 1 through peak 6; (/st/, /pət/, and /pt/ word types: peak 1 through peak 4), (2) attention modulation (without task, with task), (3) order of condition presentation (attend 2, attend 1), and (4) language experience (English, Polish) on peak latencies for each word type, separately. Comparison of a random slope MEM with a random intercept MEM found a significant difference between models for only the /pət/ word type (*p* < 0.05). The random slope model, which examined the interaction of the fixed effects of peak, attention, order and language with random effects for subject and the subject by attention interaction was found to be a better model for the /pət/ word type. Thus, the random slope model was used to analyze data. Also, testing the interaction of the fixed effects, rather than adding the fixed effect variables within the random slope model, better explained the data. Reference categories for each word type were “peak 1,” “with task,” “attend 2” (“without task” as the first testing session and “with task” as the second testing session) and “English.” The fixed effect coefficient estimate for each variable was interpreted as the effect of that variable when all other variables are zero or at reference level (categorical variables). For example, the variable English had two levels with English as the reference. The coefficient for language indicates the difference in peak latency between English and Polish subjects, when the other variables are at their reference level or at zero. Tukey HSD *post-hoc* testing followed MEM random slope analysis.

Peak latencies within individual subject's P1-N1-P2 waveforms were first identified as the highest (or lowest) amplitude value within 50 ms pre- and post-grand mean peak latency. Individual subject's waveforms were then reviewed manually to verify peak latencies. If noise in individual subject's waveforms prevented certainty for identification of a peak within the specified time interval, the averaged latency from two or, on rare occasions, three peaks within the time interval was used as the peak latency. We were unable to statistically analyze the effects of task-related attention and language experience on the T-complex morphology because high inter-subject variability in T-complex waveforms did not permit identification of peak latencies for many subjects.

##### The effects of task-related attention and language experience on AEP waveforms

To test our prediction that attention modulation would affect the AEPs by generating positive or negative shifts in the waveforms similarly in English and Polish listeners, we assessed the effects of task-related attention modulation (without task, with task), order of condition presentation (attend 2, attend 1) and language experience (English, Polish) on amplitudes obtained from the averaged fronto-central (i.e., 4, 5, 55, Cz) and posterior temporal electrodes sites (i.e., 24, 52) to each of the four word types, separately. Time intervals between 50 and 900 ms were analyzed to include exogenous processing of the stimulus attributes (Wagner et al., 2013, 2016) as well as potential endogenous processing related to task performance (Nourski et al., 2015). A random intercept MEM that controlled for individual subject's means was first compared with a random slope MEM. A random slope model using the fixed effects of attention, language and order and random effects for subject and the subject by attention interaction was found to be a better model for the data than a ME (mixed effect) random intercept model. Analyses using the random slope model in which fixed effects were added to the model and in which the interaction of fixed effects were assessed found similar results. Thus, we report only the effects for the random slope interaction model. Tukey HSD *post-hoc* analysis was used to explain significant results from the MEM random slope analyses.

Amplitudes were averaged in 50 ms intervals to each of four word types (i.e., /pt/, /pət/, /st/ /sət/). MEM analysis allows for nesting of subjects' single trial AEP responses within the individual subject data, thus, ~50,000 single trials included for analysis were nested within 48 subjects. Outliers >99.75 percentile and <0.25 percentile of the data were eliminated prior to analysis.

Global field power was calculated by obtaining the standard deviation of amplitude values from all 63 electrodes (excluding eye channels 63 and 64) at each data point between −100 and 900 ms. Current source density (CSD) maps were created using BESA (version 5.3.7).

## Results

The AEP morphology elicited by naturally spoken words was not modulated by experimental task conditions or language experience. Task-related attention, however, resulted in amplitude shifts within the fronto-central AEP that was in turn modulated by language experience. The effects of attention on the AEP from bilateral posterior temporal sites were minimal.

### Spectro-temporal processing of spoken words

Consistency of the fronto-central AEP morphology for attention-modulating tasks and language experience was assessed by examining the effects of peak (i.e., first peak, second peak etc.), task-related attention, order of condition presentation, and language experience on the latency for each positive or negative peak within the waveforms. Consistent peak latencies for task conditions in English and Polish listeners were found to three out of four phoneme sequence onsets examined, including an onset sequence (i.e., /pt/) that occurs in only one of the two languages. The latency for one peak (i.e., peak 4) within the AEP elicited to the /pət/ onset was affected by task (coefficient 22.278 ms, SEM 8.738, *p* < 0.05), however, this effect was not significant on Tukey HSD *post-hoc* testing. Thus, the morphology of the sensory AEP elicited by naturally spoken words was similar for tasks that modulated attention in English and Polish listeners. A significant main effect of peak (*p* < 0.001) for each word type, which indicated that latencies clustered around the peaks (first peak, second peak etc.), further supported this conclusion.

We were unable to statistically test the effects of task-related attention and language experience on the T-complex peak latencies, however, Figure 1 illustrates that grand mean P1-N1-P2 and T-complex morphologies were similar to the same phoneme sequence onset (e.g., /sət/) (B) for the “with task” and (C) “without task” conditions. That is peak latencies, which reflect the change in positive (or negative) direction within the AEP were similar for the P1-N1-P2 and T-complex to the same phoneme sequence onset.

### The effects of attention on the fronto-central AEP

**Figure 2:**
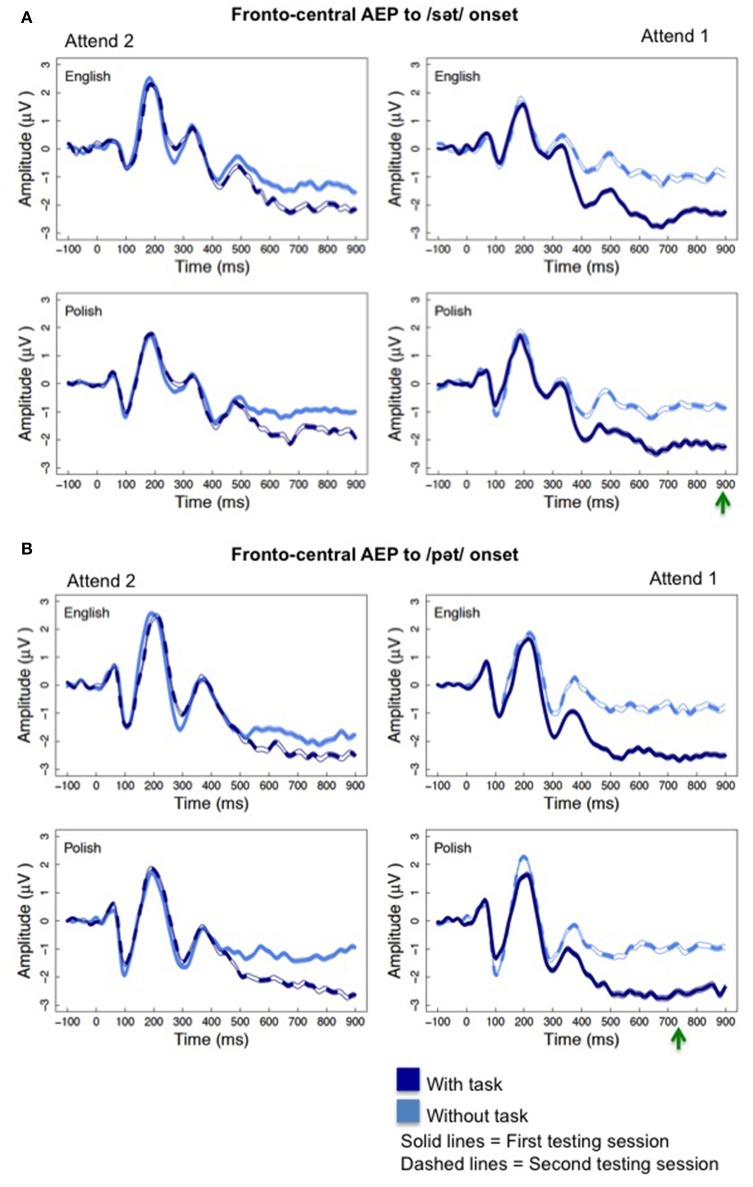
The effect of attention on the fronto-central AEP **(A)** to the /s/ onset and **(B)** the /p/ onset. Notice the negative-going deflection for the “with task” conditions (dark blue, solid and dotted lines) relative to the “without task” conditions (light blue, solid and dotted lines) that was more pronounced when the “with task” condition occurred during the first testing session (dark blue, solid lines) within the “Attend 1” sequence of condition presentation (right). The green arrows depict the earliest possible appearance for the second word in the word pairs.

**Figure 3:**
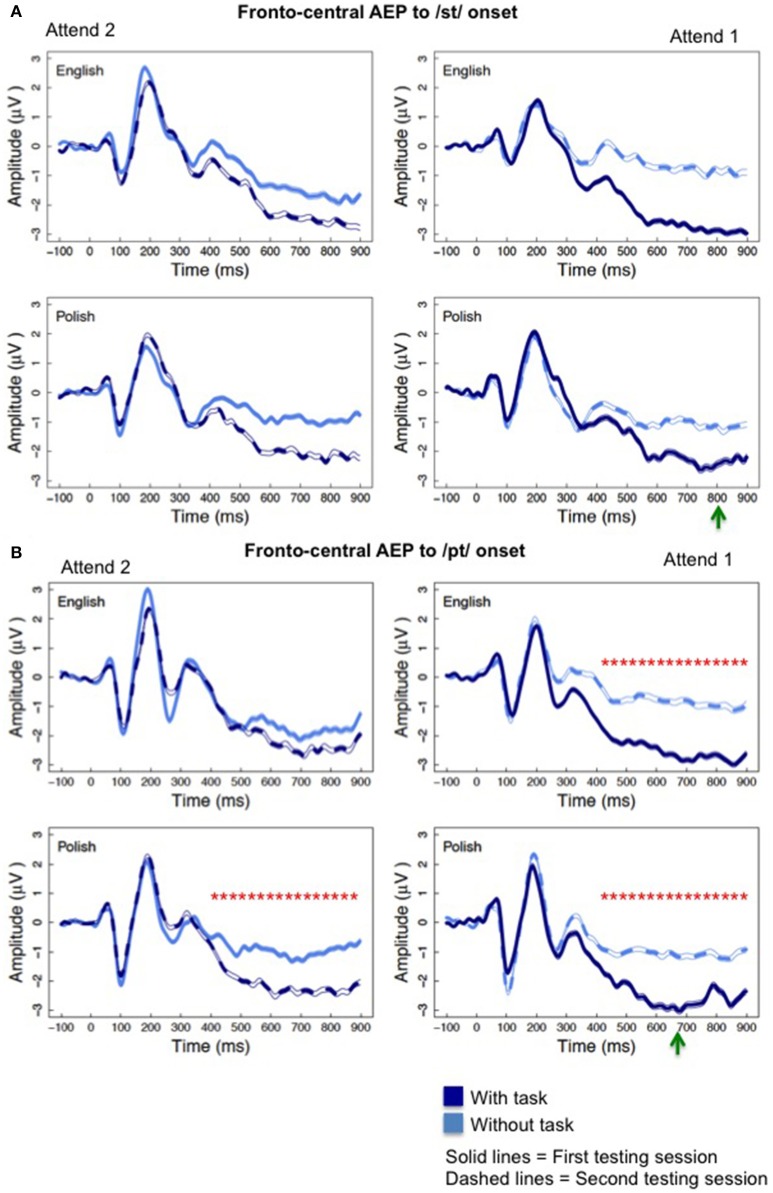
The effect of attention on the fronto-central AEP **(A)** to the /st/ onset. Notice the negative-going deflections for the “with task” conditions (dark blue, solid and dotted lines) relative to the “without task” conditions that was more pronounced when the “with task” condition occurred during testing session 1 (dark blue, solid lines) within the “Attend 1” sequence of condition presentation (right). **(B)** Graphs illustrate the significant effect of attention on the fronto-central AEP between 400 and 900 ms that occurred to the /pt/ onset cluster for the Polish participants, but not the English participants, in the “Attend 2” presentation groups (left). The green arrows depict the earliest possible appearance for the second word in the word pairs.^*^Significant effects of attention as described within text.

Attention affected the AEP at averaged fronto-central electrode sites evidenced by a negative-going shift in the waveform. Further, language experience modulated this effect of attention. Figure 2 illustrates the effects of attention on the fronto-central AEP to the /sət/ and /pət/ onsets and Figure 3 illustrates the effects to the /st/ and /pt/ onsets. For each word type, AEP amplitudes were more negative as participants were cued by presentation of the first word to attend to the task-relevant upcoming word. Green arrows in Figures 2, 3 depict the earliest possible presentation for the second word in the word pairs. The latency for presentation of the second word in the word pairs differed for each phoneme sequence as word duration varied for the naturally produced stimuli. The significant effects of attention and significant interactions involving attention described in detail below occurred even after the onset of the second word, which began as early as ~700 ms for the /pt/ onsets.

Mixed effect random slope model analysis examined the interaction of attention, order of condition presentation, and language experience on fronto-central AEP amplitudes to each word type, separately. For the /pət/ and /pt/ word types, significant main effects of attention and/or significant interactions of attention and order were found between 250 and 900 ms with a more negative waveform to the stimuli for the “with task” condition relative to the “without task” condition. For the /sət/ and /st/ word types, these significant effects began 100 ms later, occurring between 350 and 900 ms. Table 2 displays these significant main effects of attention and significant attention and order interactions, providing the coefficient estimates (standard error of mean) and the *p*-values for the /pət/, /pt/, /sət/, and /st/ word types, respectively. The 100 ms delay for the significant attention effect, as well as for the attention by order effect, for the /sət/ and /st/ onsets relative to the /pət/ and /pt/ onsets, which is consistent with a 100 ms increase in mean duration for the /sət/ and /st/ relative to the /pət/ and /pt/ onsets, was confirmed by the Tukey HSD *post-hoc* analysis. Also, consistent with the significant interactions of attention and order of task presentation, Tukey HSD *post-hoc* testing revealed that the attention effect began later at 350 ms for the “Attend 2” participant groups, who performed the attention task during session two, and the effect was elicited at fewer time intervals than for participants in the “Attend 1” order of presentation. Figures 2, 3 (left graphs) illustrate this reduced effect of attention for the “Attend 2” participant groups, who had prior experience with the stimuli and the experimental protocol.

**Table 2:** The significant main effects of attention and the significant interactions between attention and order for condition presentation (MEM random slope analysis) in response to (A) /pet/ and /pt/ word types between 250 and 900 ms and (B) /set/ and /st/ word types between 350 and 900 ms.

**(A)**	**Attention /pət/**	Attention ^*^ Order /pət/	**Attention /pt/**	Attention ^*^ Order /pt/
250	−0.391 (0.184) (<0.05)^*^	1.098 (0.258) (<0.001)^*^	−0.439 (0.430) (0.05)	1.245 (0.311) (<.001)^*^
300	−0.117 (0.207) (0.57)	0.959 (0.291) (<0.01)^*^	0.158 (0.204) (0.442)	0.569 (0.286) (<0.05)^*^
350	0.023 (0.228) (0.10)	1.130 (0.320) (<0.01)^*^	−0.031 (0.202) (0.88)	1.143 (0.284) (<.001)^*^
400	0.172 (0.249) (0.49)	1.337 (0.351) (<0.001)^*^	0.007 (0.208) (0.97)	1.301 (0.292) (<0.001)^*^
450	0.210 (0.262) (0.43)	1.261 (0.369) (<0.01)^*^	0.154 (0.244) (0.53)	1.247 (0.344) (<0.001)^*^
500	0.428 (0.279) (0.13)	1.240 (0.393) (<0.01)^*^	0.139 (0.255) (0.59)	1.289 (0.360) (<0.001)^*^
550	0.881 (0.293) (<0.01)^*^	0.679 (0.413) (0.11)	0.425 (0.250) (0.10)	1.153 (0.351) (<0.01)^*^
600	0.799 (0.305) (<0.05)^*^	0.639 (0.430) (0.15)	0.589 (0.287) (<0.05)	1.058 (0.405) (<0.05)^*^
650	0.916 (0.300) (<0.01)^*^	0.874 (0.422) (<0.05)^*^	0.509 (0.270) (0.07)	1.383 (0.380) (<0.001)^*^
700	0.713 (0.318) (<0.05)^*^	1.059 (0.447) (<0.05)^*^	0.500 (0.285) (0.09)	1.328 (0.402) (<0.01)^*^
750	0.742 (0.294) (<0.05)^*^	0.866 (0.414) (<0.05)^*^	0.595 (0.268) (<0.05)	1.078 (0.377) (<0.01)^*^
800	0.853 (0.276) (<0.01)^*^	0.824 (0.388) (<0.05)^*^	0.708 (0.278) (<0.05)	0.985 (0.392) (<0.05)^*^
850	0.923 (0.30) (<0.01)^*^	0.715 (0.423) (0.10)	0.670 (0.262) (<0.05)	0.962 (0.369) (<0.05)^*^
**(B)**	**Attention /sət/**	Attention ^*^ Order /sət/	**Attention /st/**	Attention ^*^ Order /st/
250	−0.3767 (0.20) (0.07)	0.5437 (0.281) (0.06)	−0.186 (0.203) (0.36)	0.421 (0.285) (0.15)
300	0.049 (0.20) (0.81)	0.275 (0.282) (0.33)	0.117 (0.203) (0.57)	0.292 (0.285) (0.31)
350	0.103 (0.199) (0.605)	0.886 (0.280) (<0.01)^*^	−0.617 (0.286) (<0.01)^*^	0.190 (0.279) (0.50)
400	0.249 (0.209) (0.24)	1.039 (0.294) (<0.001)^*^	0.681 (0.214) (<0.01)^*^	0.403 (0.301) (0.19)
450	0.316 (0.233) (0.18)	0.813 (0.328) (<0.05)^*^	0.763 (0.249) (<0.01)^*^	0.473 (0.350) (0.18)
500	0.416 (0.237) (0.09)	0.815 (0.334) (<0.05)^*^	0.570 (0.246) (<0.05)^*^	1.072 (0.346) (<0.01)^*^
550	0.610 (0.240) (<0.05)^*^	0.829 (0.339) (<0.05)^*^	0.828 (.217) (<0.001)^*^	0.958 (0.305) (<0.01)^*^
600	0.613 (0.257) (<0.05)^*^	0.966 (0.362) (<0.05)^*^	0.997 (0.224) (<0.001)^*^	0.768 (0.316) (<0.05)^*^
650	0.698 (0.277) (<0.05)^*^	0.912 (0.390) (<0.05)^*^	0.942 (0.228) (<0.001)^*^	0.870 (0.320) (<0.01)^*^
700	0.822 (0.273) (<0.01)^*^	0.601 (0.384) (0.13)	0.807 (0.244) (<0.01)^*^	1.213 (0.344) (<0.001)^*^
750	0.760 (0.254) (<0.01)^*^	0.449 (0.358) (0.22)	0.928 (0.253) (<0.001)^*^	1.043 (0.356) (<0.01)^*^
800	0.819 (0.267) (<0.01)^*^	0.314 (0.276) (0.41)	0.695 (0.253) (<0.01)^*^	1.378 (0.356) (<0.001)^*^
850	0.791 (0.242) (<0.01)^*^	0.575 (0.341) (0.10)	0.892 (0.264) (<0.01)^*^	1.031 (0.372) (<0.01)^*^

*Coefficients (SEM) (p-values) are provided*.

* *Significant effects*.

**Figure 4:**
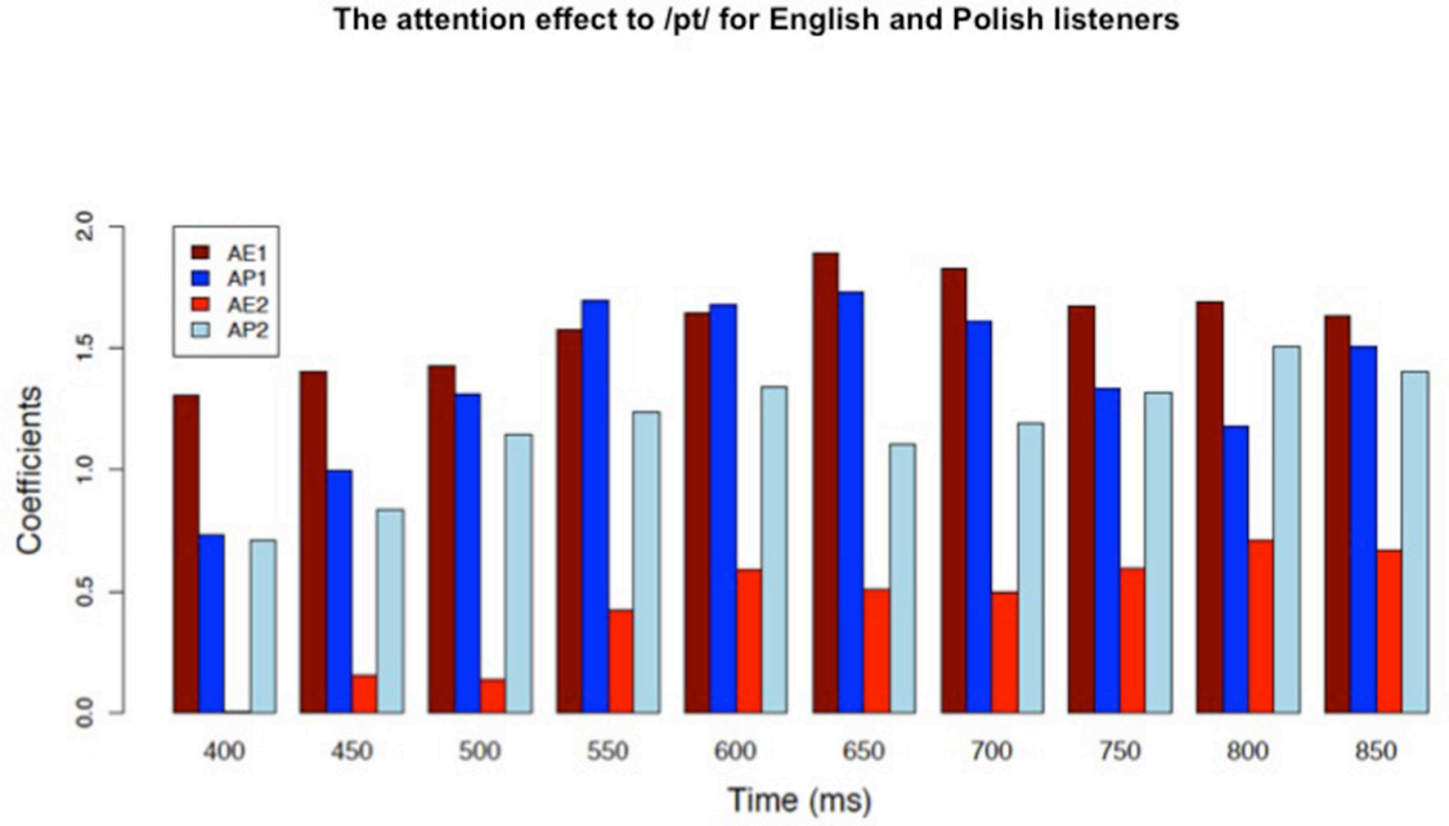
A significant attention effect (Tukey HSD) between 400 and 900 ms to the /pt/ onset was found for Polish listeners (light blue bar), but not English listeners (red bar) when the attention task was presented during testing session two (AP2, AE2). Note that the /pt/ onset is a familiar phonotactic pattern for only the Polish language group. The dark blue and brown bars indicate the significant attention effect found for both language groups to /pt/ onset when the attention task was presented during testing session one (AP1, AE1).

As illustrated in Table 3, a consistent interaction of attention and language experience was found between 400 and 900 ms for only the /pt/ phoneme sequence onset, which occurs in the Polish language, but not the English language. Also, notice in Table 3, that the significant interaction of language and attention was not found to the /st/, /sət/, or /pət/ onsets that occur in both the English and Polish languages. The significant interaction was explained by Tukey HSD testing, which revealed that a significant effect of attention was found for the Polish participants, but not the English participants to the /pt/ onset between 400 and 900 ms for the “Attend 2” presentation group (Figure 4). Effects of attention to the phoneme sequence onsets (i.e., /st/, /pət/, and /sət/) that occur in both languages did not differ for English and Polish listeners for this task presentation order. Also, language and attention did not interact for any word types for participants receiving the “Attend 1” presentation sequence.

**Table 3:** The significant interactions between attention and language experience (MEM random slope analysis) in response to only the /pt/ onset that occurs in the Polish language, but not the English language, between 400 and 900 ms.

	**/pet/**	**/pt/**	**/set/**	**/st/**
400	−0.051 (0.246) (0.84)	0.703 (0.245) (<0.01)^*^	−0.201 (0.248) (0.42)	−0.274 (0.244) (0.26)
450	0.139 (0.247) (0.58)	0.680 (0.248) (<0.01)^*^	−0.202 (0.253) (0.42)	−0.102 (0.248) (0.68)
500	0.099 (0.254) (0.70)	1.010 (0.257) (<0.001)^*^	−0.152 (0.255) (0.55)	0.303 (0.253) (0.23)
550	0.113 (0.256) (0.66)	0.811 (0.262) (<0.01)^*^	−0.097 (0.259) (0.71)	0.235 (0.256) (0.36)
600	−0.063 (0.261) (0.81)	0.753 (0.263) (<0.01)^*^	0.041 (0.262) (0.87)	0.002 (0.259) (0.99)
650	−0.039 (0.263) (0.88)	0.595 (0.268) (<0.05)^*^	0.108 (0.263) (0.68)	0.175 (0.262) (0.50)
700	0.077 (0.264) (0.77)	0.694 (0.269) (<0.01)^*^	−0.253 (0.266) (0.34)	0.336 (0.263) (0.20)
750	0.297 (0.269) (0.27)	0.725 (0.272) (<0.01)^*^	−0.091 (0.269) (0.74)	0.182 (0.268) (0.50)
800	0.336 (0.267) (0.21)	0.802 (0.274) (<0.01)^*^	−0.075 (0.271) (0.78)	0.341 (0.271) (0.21)
850	0.619 (0.274) (<0.05)	0.736 (0.278) (<0.01)^*^	−0.073 (0.275) (0.79)	0.365 (0.271) (0.18)

*This effect was not found for the /pet/, /set/ or /st/ onsets. For each time interval, the coefficient (SEM) (p value) is provided*.

* *Significant effects*.

**Figure 5:**
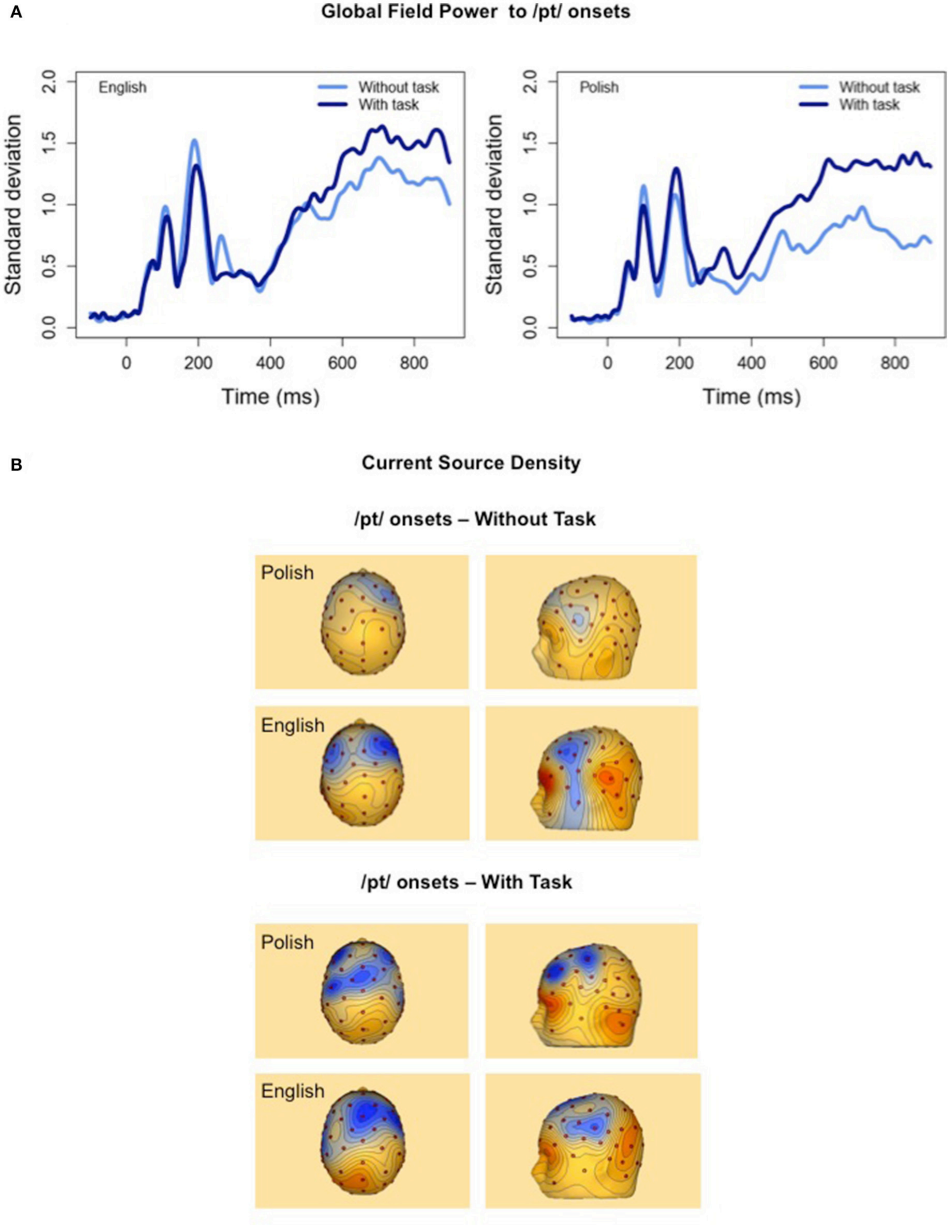
**(A)** Global Field Power graphs to the /pt/ onset in English and Polish participants that support the significant interaction between attention and language experience between 400 and 900 ms found for the fronto-central AEP (MEM analysis). **(B)** Current Source Density maps at 600 ms to the /pt/ onset support the significant attention effect found for the “with task” condition (bottom) relative to the “without task” condition (top) for the Polish participants, but not the English participants (Tukey HSD). Notice that processing patterns differ for the language groups for the “without task” condition to the /pt/ onsets. English and Polish participants in the Attend 2 sequence of presentation are shown.

Global Field Power (GFP) waveforms and CSD maps to the /pt/ onset in English and Polish listeners in the “Attend 2” groups are shown in Figure 5. GFP graphs supported MEM and Tukey HSD *post-hoc* results that found a significant interaction of language and attention at averaged fronto-temporal sites, with a significant effect of attention for only the Polish listeners to the /pt/ onset between 400 and 900 ms (Figure 5A). CSD maps in Figure 5B to the /pt/ onset at 600 ms revealed a greater fronto-central response for the “without task” condition for the English relative to the Polish listeners.

### The effects of attention on the posterior temporal AEP

The effect of attention on the posterior temporal AEP was minimal. A significant effect of attention to the /st/ word type was found on *post-hoc* analysis for only the English listeners (“Attend 1”) at 400 ms and between 500 through 650 ms.

This result is ambiguous because a significant interaction of attention and language at these time intervals to the /st/ word type was not found on MEM analysis, therefore, this result should be considered with caution.

### Early processing effects on the P1-N1-P2

*Post-hoc* analyses of the P1-N1-P2 response also revealed a greater degree of negative amplitudes for the Polish listeners relative to the English listeners in the “without task” conditions (e.g., Polish response more negative than English response at 150 ms to /set/ onset) that were significant at some time intervals between 100 and 450 ms as detailed below. These effects were not specific to word type. Interestingly, the bilingual Polish-English participants showed the more negative responses relative to the monolingual English participants.

Specifically, a greater negative amplitude for the Polish participants relative to the English participants was significant on *post-hoc* testing for the “Attend 2” groups at 150 for all word types (/pət/ coefficient −0.925, SEM 0.151, *p* < 0.001; /pt/ coefficient −0.631, SEM 0.151, *p* < 0.001; /sət/ coefficient −0.735, SEM 0.151, *p* < 0.001; /st/ coefficient −0.933, SEM 0.149, *p* < 0.001), at 200 ms for /st/ (coefficient −0.535, SEM 0.153, *p* < 0.01) and /sət/ word types (coefficient −0.496, SEM 0.156, *p* < 0.05), and at 300 ms for /pət/ (coefficient −0.589, SEM 0.164, *p* < 0.01) and /st/ word types (coefficient −0.558, SEM 0.142, *p* < 0.01). The effect was also significant for the “Attend 1” group at 100 ms for /pt/ (coefficient −0.508, SEM 0.144, *p* < 0.01) and /st/ (coefficient −0.545, SEM 0.164, *p* < 0.05), at 300 ms for /st/ (coefficient −0.599, SEM 0.166, *p* < 0.01), and at 450 ms for /pət/ (coefficient −0.532, SEM 0.176, *p* < 0.05).

### Early processing effects on the T-complex

The T-complex showed an inverse response relative to the P1-N1-P2 with the Polish listeners showing more positive amplitudes than the English listeners during some time intervals. The response was always more positive for the Polish listeners relative to the English listeners and was not specific for word type. In contrast to early stage effects at the fronto-central sites, the increased positivity overlying PLST was found for both the “without task” (“Attend 2”) and “with task” (“Attend 2”) conditions.

For example, in response to the /pet/ onset, *post-hoc* testing revealed that the Polish listeners showed significantly more positive amplitudes for the “without task” (“Attend 2”) and “with task” (“Attend 2”) conditions relative to the English listeners at 150 ms (coefficient 0.497, SEM 0.108, *p* < 0.001; coefficient 0.377, SEM 0.12, *p* < 0.05) and at 200 ms (coefficient 0.647, SEM 0.115, *p* < 0.001; coefficient 0.615, SEM 0.118, *p* < 0.001) and at 300 ms for the “without task” condition (“Attend 2”) only (coefficient 0.407, SEM 0.135, *p* < 0.05). In response to the /set/ onset, the Polish listeners showed significantly more positive amplitudes for both “without task” (“Attend 2”) and “with task” conditions (“Attend 2”) at 100 (coefficient 0.355, SEM 0.098, *p* < 0.01; coefficient 0.317, SEM 0.10, *p* < 0.05) and 150 ms (coefficient 0.452, SEM 0.109, *p* < 0.001; coefficient 0.434, SEM 0.111, *p* < 0.01), at 200 ms for the “with task” condition (Attend 2) [coefficient 0.383, (0.121), *p* < 0.05] and at 300 ms for the “without task” condition (“Attend 2”) only (coefficient 0.564, SEM 0.137, *p* < 0.001). In response to the /pt/ word type, significantly more positive amplitudes were found for the “with task” condition (“Attend 1”) for the Polish listeners relative to the English listeners at 50 ms (coefficient 0.279, SEM 0.085, *p* < 0.05) and 200 ms (coefficient 0.441, SEM 0.118, *p* < 0.01).

## Discussion

The current study revealed that the morphology of the P1-N1-P2 was similar for tasks that modulated attention and language experience. This stability suggests that the P1-N1-P2 that reflects cortical level recognition of spectro-temporal feature changes within the spoken word might be useful in probing auditory processing deficits in individuals with language impairment and comorbid attentional deficits. The study also revealed that task-related attention resulted in a negative-going shift in the fronto-central AEP response between 400 and 900 ms that was modulated by prolonged experience with a phoneme sequence. Hence, attention appears to enhance native-language features within spoken words at intermediate stages within auditory cortex, which may facilitate word recognition for comprehension.

### Spectro-temporal feature processing

The P1-N1-P2 and T-complex to the phoneme sequence onsets used in the current study are elicited between 50 and 600 ms with spectral and durational differences between the phoneme sequences (e.g., /pət/ vs. /sət) reflected in the waveform morphology (Wagner et al., 2013, 2016). The morphology of the P1-N1-P2, specific for each phoneme sequence and characterized by a series of peak latencies, was not affected by native-language, task condition, or sequence of condition presentation. Also, P1-N1-P2 peak latencies in response to the /pt/ onsets, which cannot be distinguished from /pət/ onsets by English listeners (Wagner et al., 2012), were not influenced by language experience. Thus, the current study provided additional support for the view that the P1-N1-P2 reflects acoustic, rather than linguistic processing of speech (Sharma and Dorman, 2000; Elangovan and Stuart, 2011; Wagner et al., 2013). T-complex peak latencies also appeared consistent for native-language, task condition and sequence of condition presentation on visual inspection of waveforms, however, inter-subject variability in T-complex waveforms prevented statistical analysis.

Attention did not alter representation of spectro-temporal feature processing, which support the findings of Nourski et al. (2015) who examined the EEG recorded directly from PLST (posterior lateral superior temporal area). An effect of attention on high gamma responses to a target stimulus in a stream of non-target stimuli did not modulate representation of the acoustic attributes of the stimulus, however, the timing of the effect of attention was consistent with subsequent sound processing. Thus, consistent with Nourski and colleagues, our results suggest that acoustic characteristics within spoken words are enhanced for linguistic processing at intermediate stages within auditory cortex. These researchers also found no effect of attention within the AEPs overlying PLST, which is consistent with our result for the T-complex.

### The attention effect on the P1-N1-P2 waveform

An effect of attention for task performance was found for the fronto-central AEP, but not for the posterior-temporal AEP, for all word types. The effect began between 250 to 350 ms, and peaked for a long duration between 400 and 900 ms. The onset of the attention effect appeared contingent on the duration of the phoneme sequences, which was consistent with other studies that used a slow rate of stimulus presentation (Hansen and Hillyard, 1984; Woldorff and Hillyard, 1991; Alain et al., 1993; Nourski et al., 2015). The attention effect in our study, however, persisted for a longer duration than in other studies (Woldorff and Hillyard, 1991), at least through 900 ms, even after the onset of the subsequent word. In contrast to other experiments that investigated the response to target stimuli randomly presented among non-target stimuli, our participants were aware that for experimental conditions that included a behavioral task, the non-target first word prompted the occurrence of the target word. We are unaware of other studies having a similar experimental design that examined the effects of attention on auditory sensory processing. The current results suggested heightened vigilance for the first word in the word pairs for the attention conditions, as well as heightened vigilance that continued beyond the duration of the first words, which might facilitate processing of the subsequent word, necessary to perform the task. Within the context of conversation, one prepares to listen to a particular word to facilitate recognition of that word. Based on the current results, it is possible that networks within auditory cortex enhance feature processing in preparation for recognition of a key word within a passage.

The attention effect began 100 ms earlier for the /pt/ and /pət/ onsets relative to the /st/ and /set/ onsets, which is consistent with the differences in the mean duration of the phoneme sequence onsets. Thus, the timing for the occurrence of the second word, the target, could be predicted by the occurrence of the first word in the word pairs. Words within the pairs began with either a short duration stop phoneme (i.e., /p/) or a long duration fricative phoneme (i.e., /s/) and different word pairs contained only the addition of a schwa (e.g., /ptimɑ-pətimɑ/, /stimɑ-sətimɑ/). Thus, it is possible that the regularity of the input (i.e., mean duration of the phoneme sequences in the first words) provided the timing for the occurrence of the subsequent word, which interacted with vigilance in preparation to perform the task. Stimulus timing has been shown to facilitate sensory processing within visual cortex (Schroeder and Lakatos, 2008; Rohenkohl and Nobre, 2011; Rohenkohl et al., 2012). For example, Rohenkohl and Nobre (2011) demonstrated that the timing of visual stimulus presentations modulated alpha band oscillations in preparation for a subsequent visual target, which the authors suggested might enhance processing of the target stimulus.

### The attention effect enhanced by experience with a phoneme sequence

Our prediction that attention would affect the AEPs to all phoneme sequences similarly in English and Polish listeners was not supported. In contrast, the effect of attention on the fronto-central waveform was influenced by native-language experience with the /pt/ phoneme sequence onset that occurs only in the Polish language. For example, the Polish word for “bird” begins with the /pt/ consonant cluster, however, the English language has no spoken words that begin with the /pt/ consonant cluster. Thus, English listeners never hear the /pt/ cluster without a preceding vowel. The attention effect to the /pt/ onset for only the Polish listeners was elicited when the behavioral task occurred during the second testing session (i.e., “Attend 2”), thus, participants were previously exposed to the non-words and the testing protocol during their first session. Performing the behavioral task as the second testing session (i.e., “Attend 2”), rather than the first testing session (i.e., “Attend 1”), resulted in later and fewer significant effects of attention. This lessened effect of attention, suggests that task performance demands were reduced during session two that occurred two or more months after session one. Examining the effects of attention to speech within the mismatch negativity response (MMN) of the AEP, Hisagi et al. (2010) demonstrated that only when attention was directed away from the speech stimulus did native and non-native listeners show different processing patterns. Thus, comparison of responses within the “without task” and “with task” conditions in the “Attend 2” sequence of presentation, with reduced effects of attention, may have unmasked automatic patterns of native-language processing (Strange, 2011). In contrast, the alternate group of English and Polish subjects who performed the behavioral task during testing session 1 (“Attend 1”) showed larger effects of attention to all phoneme sequences. The more prominent negativity for this group may reflect an increased readiness response (Walter et al., 1964) originating in motor/premotor cortex, possibly concealing linguistic processing differences associated with vigilance. Interestingly, the early stage processing effect within the P1-N1-P2 that found the bilingual Polish-English listeners to have more negative responses than the monolingual English listeners that was not specific to word type occurred only for the “without task” conditions, which required less vigilance than the “with task” conditions. Irrespective of the cause of the reduced attention effect for subjects participating in the “Attend 2” sequence for condition presentation, the consistent difference between the English and Polish listeners in response to only the phoneme sequence onset /pt/ that persisted for at least 500 ms suggests an enhancement of native-language features of speech for linguistic level processing at intermediate stages within auditory cortex.

### Early stage processing effect

An early stage processing effect modulated the P1-N1-P2 in the form of increased negative amplitudes for the bilingual Polish listeners relative to the monolingual English listeners. The T-complex showed a similar processing effect with increased positive responses for the bilingual Polish listeners relative to the English listeners. It is possible, however, that T-complex responses reflected inverted activity from generators on the superior temporal plane. This early cortical stage response was not specific to any particular word type. Therefore, the nature of engaging in two languages might enhance sensory processing within auditory cortex. Consistent with the view of enhanced sensory processing in bilinguals, work by Kriznan et al. (2012) revealed enhanced processing to speech at subcortical levels in bilinguals relative to monolingual subjects. Also, Vihla et al. (2002) using magnetoencephalography (MEG) found general auditory processing differences in bilingual speakers of Finnish and Swedish relative to monolingual Finish speakers with right hemisphere processing differences (~200 ms) found in response to tone stimuli, as well as vowel stimuli. Also, Tamminen et al. (2013) found different neural patterns for the MMN response to native-language sound contrasts in monolingual subjects relative to balanced bilingual subjects who learned two languages beginning at birth. The authors argued that the two native languages including their phonologies were active in the balanced bilinguals for neural speech processing.

It is also possible that the native-Polish speakers in our study, who learned English as young adults, engaged increased attention within the English-speaking environment (e.g., instructions presented in English) relative to the English participants. It will be interesting to examine this possibility in future studies.

### Future clinical and research application of P1-N1-P2 and T-complex

The current results have significance for future studies examining auditory processing deficits in individuals with language impairment and comorbid attentional deficits, such as individuals with Autism and Specific Language Impairment (Ceponiene et al., 2003; Stevens et al., 2006; Shafer et al., 2007; O'Conner, 2012; Orekhova and Stroganova, 2014). Using a similar experimental design, the morphology of the P1-N1-P2 might be used to assess recognition of spectro-temporal feature changes within the spoken word within auditory cortex (Giraud et al., 2005; Wagner et al., 2016) as P1-N1-P2 remained consistent for attention-related task performance. Additional studies will be necessary to support the use T-complex waveforms for probing auditory processing deficits in clinical populations known to have associated attentional deficits.

Also, understanding the neural processes that facilitate recognition of a subsequent word within conversation has translational relevance as individuals with autism have an impaired ability to disengage and engage attention to a target stimulus (Ceponiene et al., 2003; Orekhova and Stroganova, 2014). Hence, it will be interesting to learn in future studies whether preparatory vigilance, identified in the current study, can be demonstrated in individuals with Autism. It is possible that an impaired ability to “prepare” to attend to select words within a passage can partially explain auditory processing deficits (O'Conner, 2012) in this population.

## Ethics statement

Research protocols were approved by The Graduate Center, City University of New York's Internal Review Board and St. John's University's Internal Review Board. All subjects gave written informed consent in accordance with the Declaration of Helsinki.

## Author contributions

MW, MS, and VS conceived and designed the experiment. MW conducted the study from data collection and analysis through writing the manuscript. MW and MS wrote the manuscript. MS assisted in interpretation of data. JL and AC wrote computer scripts for data analysis, analyzed, and interpreted data. CO and FM performed data processing and analysis. All authors reviewed and edited the manuscript.

### Conflict of interest statement

The authors declare that the research was conducted in the absence of any commercial or financial relationships that could be construed as a potential conflict of interest.
